# Pedagogical monitoring as a tool to reduce dropout in distance learning in family health

**DOI:** 10.1186/s12909-016-0735-9

**Published:** 2016-08-22

**Authors:** Deborah de Castro e Lima Baesse, Alexandra Monteiro Grisolia, Ana Emilia Figueiredo de Oliveira

**Affiliations:** 1 Universidade Aberta do Sistema Único de Saúde - UNA-SUS / Universidade Federal do Maranhão - UFMA, Rua Viana Vaz, 41, Centro, 65020-660 São Luís, Maranhão Brasil; 2 Centro Biomédico, Faculdade de Ciências Médicas, Universidade do Estado do Rio de Janeiro, Av.Vinte e Oito de Setembro, 77, Sala 126, Térreo, Vila Isabel, Rio de Janeiro, 20561-030 Rio de Janeiro Brazil

**Keywords:** Dropout, Distance learning, Pedagogical support, Specialization

## Abstract

**Background:**

This paper presents the results of a study of the Monsys monitoring system, an educational support tool designed to prevent and control the dropout rate in a distance learning course in family health. Developed by UNA-SUS/UFMA, Monsys was created to enable data mining in the virtual learning environment known as Moodle.

**Methods:**

This is an exploratory study using documentary and bibliographic research and analysis of the Monsys database. Two classes (2010 and 2011) were selected as research subjects, one with Monsys intervention and the other without. The samples were matched (using a ration of 1:1) by gender, age, marital status, graduation year, previous graduation status, location and profession. Statistical analysis was performed using the chi-square test and a multivariate logistic regression model with a 5 % significance level.

**Results:**

The findings show that the dropout rate in the class in which Monsys was not employed (2010) was 43.2 %. However, the dropout rate in the class of 2011, in which the tool was employed as a pedagogical team aid, was 30.6 %. After statistical adjustment, the Monsys monitoring system remained in correlation with the course completion variable (adjusted OR = 1.74, IC95% = 1.17–2.59; *p* = 0.005), suggesting that the use of the Monsys tool, isolated to the adjusted variables, can enhance the likelihood that students will complete the course. Using the chi-square test, a profile analysis of students revealed a higher completion rate among women (67.7 %) than men (52.2 %). Analysis of age demonstrated that students between 40 and 49 years dropped out the least (32.1 %) and, with regard to professional training, nurses have the lowest dropout rates (36.3 %).

**Conclusions:**

The use of Monsys significantly reduced the dropout, with results showing greater association between the variables denoting presence of the monitoring system and female gender.

## Background

The process of teaching and learning is a complex phenomenon. Educational achievement requires students to access learning opportunities, to persist in their learning activities, and to complete the required stages of a course of study. When one of these links in the educational provision breaks, educational failure occurs [1].

Experts disagree on the primary causes for educational failure. Some experts view failure as an individual process, related only to the student’s ability to assimilate content. For others, it is an institutional process that results from the organizational form of the curriculum, methodologies and evaluation. Finally, for still others, it is a socio-political process that stems from context [2].

In the research described herein, educational failure is treated as a complex phenomenon involving numerous factors that contribute to “failure to learn” and for which repetition and dropout are the main manifestations [2].

Ashby [3] defines dropout as a student’s withdrawal from a course without having completed it successfully and subdivides dropout as follows: temporary interruption (stopout), exit with acquisition of knowledge (attainer), abandonment without getting started (non-starter) and real dropout (dropout). Dropout has long been an important concern among education professionals. This concern is manifest across national contexts and is present in both classroom teaching and distance learning. In our research, the focus is on the distance education (DE) modality.

As noted by Grau-Valldosera [4], Minguillón [5], Baxter [6], Fiuza [7] and Cheng et al. [8], DE dropout rates are a concern for educational institutions in general. In addition, when students start but do not finish their courses, social, academic and economic waste is generated [9–13].

Mezzari et al. [14] note that Brazil’s National Institute of Educational Studies and Research (INEP) has released data to facilitate the study of withdrawal. Based on these data, the Brazilian Statistical Yearbook of Distance Education (ABRAEAD) found that approximately one-half of the students (48 %) who annually enter the DE system in Brazil do not receive their diplomas on time [15].

DE is a flexible learning method that is based on individual autonomy and convenience of access. DE courses do not have geographic limitations and have high rates of growth [16]. The expansion of DE is of the utmost importance in the democratization of education, particularly with regard to professional development. In addition, the introduction of new information and communication technologies (ICTs) in the labor market is a factor that cannot be ignored.

Normally, DE is offered in a virtual learning environment (VLE) that is specially designed to provide support to DE students. Moodle, the platform employed by the Open University of Brazilian National Health System (UNA-SUS), boasts over 69 million users in 226 countries, including academic and business users [17].

Anticipating the numerous possibilities that DE creates for healthcare workers’ continuing education, Brazil’s federal government created UNA-SUS, a project developed by the Ministry of Health, through the Secretary of Labor Management and Health Education (SGTES) and in partnership with several institutions, including the Federal University of Maranhão (UFMA). The goal of UNA-SUS is to qualify professionals who work in the Primary Care Division of the National Health System (SUS) [18]. To that end, UNA-SUS/UFMA has continuously offered specialization courses in Family Health since 2010.

Seeking to continually improve its educational activities, UNA-SUS/UFMA developed the Monsys monitoring system as a pedagogical support tool to control dropout. The research described herein comparatively analyzes the performance of this tool in two DE classes in Family Health, one in which Monsys was not used and another in which Monsys was used. The tool, which was developed and patented by UNA-SUS/UFMA at the National Institute of Industrial Property (INPI) with registration process number BR 51 2014001542 4, uses PHP programming technologies with JAVASCRIPT and HTML and enables data mining in Moodle. The five authors of the patent are members of the UNA-SUS/UFMA team.

Moodle stores large amounts of data that are generated by continuous usage by teachers and students. However, Moodle is not a monitoring system and therefore does not organize data in relevant ways—instead, the data are dispersed among the platform pages. The Monsys tool was developed to facilitate and accelerate the collection of relevant data, information and knowledge. Its use was meant to provide more consistent results for a more successful educational process, which includes identifying unsatisfactory performance among students early enough to prevent withdrawal. Monsys helps research teams detect factors associated with dropout in DE courses, thereby contributing to better training of SUS professionals.

A review of the literature shows a gap regarding the effectiveness of pedagogical monitoring in reducing dropout [19, 20]. We define pedagogical monitoring as the systematic monitoring of students’ access to the module and their participation in the courses, the permanent monitoring of the quantity and quality of interventions made by tutors, the progress of activities, and, in particular, the rapid transformation of this information into actions that prevent dropout.

Seeking to investigate how educational management can contribute to processes that prevent dropout, our research supports the hypothesis that systematic monitoring of the teaching-learning process and prior knowledge of students’ profiles can support pedagogical actions that reduce rates of dropout in the DE context.

### The moodle platform and the monsys system

Moodle is an acronym for Modular Object-Oriented Dynamic Learning Environment [21]. It consists of open source software—allowing users to further develop and improve it—that is used primarily in virtual learning environments.

Throughout the progress of each module, Moodle produces data on the educational history of students, teachers and tutors during the course of each module. Monsys was developed and implemented to facilitate the collection of this information and to improve the courses offered by UNA-SUS/UFMA. When connected to Moodle, Monsys gathers the data collected by the platform and organizes it into a user-friendly interface that allows the pedagogical teams to monitor the activities of students and tutors in order to identify problems and facilitate progress. The main motivation in creating Monsys was to control and reduce dropout.

**Fig. 1:**
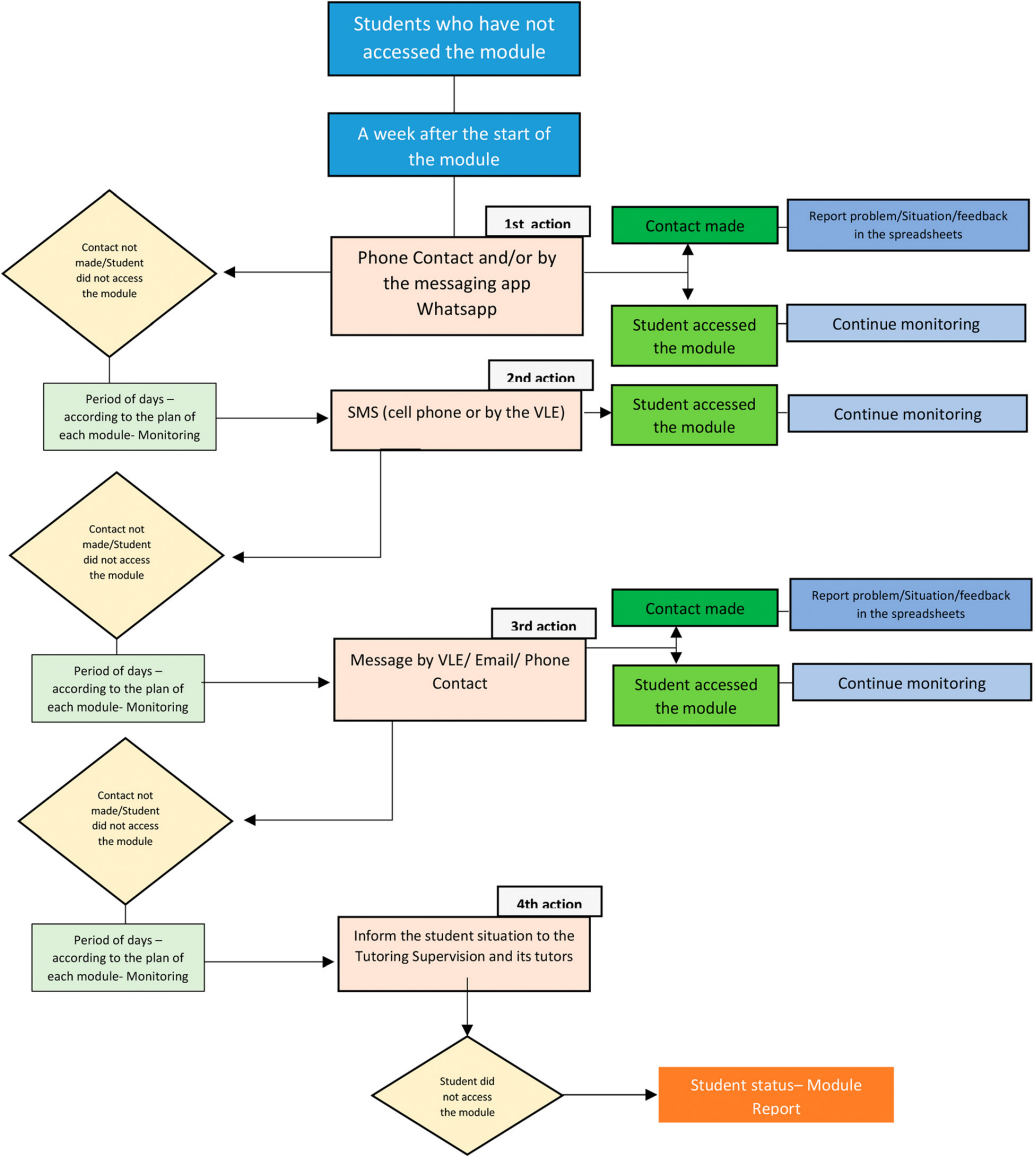
Flow of actions of the pedagogical supervision team of UNASUS/UFMA. Flow of actions established by the UNASUS/UFMA pedagogical supervision team to monitor the students who access the module infrequently or not at all

The opportunity to track students’ access to the platform is fundamental to making decisions about reducing or controlling dropout. To monitor those who access the module infrequently or not at all, the UNA-SUS/UFMA pedagogical supervision team established a flow of actions, which is shown in Fig. 1.

The results of the Monsys analysis shows that transforming data into actionable information can reduce dropout, which corroborates the findings of our research.

## Methods

The project was approved by the University Hospital Research Ethics Committee of the Federal University of Maranhão on 04/25/2014 by technical report number 641.915.

The sample consisted of students enrolled in two postgraduate classes in Family Health offered by UNA-SUS/UFMA in 2010 and 2011. One of these classes was monitored using Monsys, and the other was not. For the analysis, dropout rates were used as a comparative criterion. None of the students in either class received any type of financial incentive or financial aid. To pair the sample, we used the database from the 2010 class (*n* = 349) and 2011 (*n* = 753). The pairing was implemented considering the gender, age and profession variables at a 1:1 ratio. After pairing, beginning with the smallest class (2010), the final sample was added that was used in the study of 222 matched students from each class. The chi-square test was employed to assess the pairing.

We collected personal identification data (age, gender, marital status), professional data (occupation and place of work), course enrollment at UNA-SUS, the status of each course (graduated, failed, evaded/quit) and student participation in the course from a secondary database in the registration system of UNA-SUS/UFMA.

SPSS was used to run the statistical analysis. Descriptive statistics of the data were generated through the summary measures: absolute and relative frequencies, measures of central tendency (mean or median), dispersion measures (standard deviation or interquartile range) and inter-values estimates (confidence interval at 95 %). The results are presented in Tables 1, 2, 3, and 4.

The dependent variable or predictor in this study is the variable related to the existence of dropout (dichotomous), and the main independent variable or predictor is the presence of Monsys (dichotomous). The other independent variables are related to the student’s profile and to their performance during the activities developed in the course modules.

To test the hypothesis with the use of categorical variables, we used a chi-square test. The odds ratio (OR) measure and its confidence interval at 95 % were used to measure the association between variables. The significance level is 5 %. Multivariate logistic regression analyses were also performed considering the variables with a *p*-value less than 0.20 in the bivariate analysis. In the final model, the adjustment was carried out for the monitoring system, gender, age and profession variables.

The data collected were used for scientific purposes only, and the privacy and confidentiality of the participants involved were protected.

## Results

Table 1 describes the profile of the students in the classes that did not use Monsys as a monitoring tool (Class of 2010) and the classes that did use Monsys (Class of 2011) for the matched variables (gender, age and profession, and marital status). Females accounted for the largest percentage of the sample (69.8 %). In addition, we noted that 50.9 % of the students were between the ages of 22 and 29 and that nursing professionals comprised the largest share of analyzed students (55.9 %).

**Table 1:** Distribution of profile variables between the analyzed classes and pairing analysis

Variables	Monitoring system				*p* ^a^ value
	Absent Class 2010 (*N* = 222)		Present Class 2011 (*N* = 222)		
	F	(%)	f	(%)	
Gender					1.000
Female	155	(69.8)	155	(69.8)	
Male	67	(30.2)	67	(30.2)	
Age					1.000
22–29	113	(50.9)	113	(50.9)	
30–39	63	(28.4)	63	(28.4)	
40–49	28	(12.6)	28	(12.6)	
50 and up	18	(8.1)	18	(8.1)	
Professional training					1.000
Nursing	124	(55.9)	124	(55.9)	
Odontology	75	(33.8)	75	(33.8)	
Medicine	23	(10.4)	23	(10.4)	
Marital status					0.763
With partner	73	(32.9)	76	(34.2)	
Without a partner	149	(67.1)	146	(65.8)	

^a^Chi-square test

The association between the use of Monsys and completion of the course is described in Table 2. Notably, in the matched sample of the 2010 class (which had no monitoring system), 56.8 % of students completed the course, whereas the matched class of 2011 presented a completion rate of 69.4 %.

**Table 2:** Association between use of the monitoring system and the course completion rate

Monitoring system	Course completion				OR (IC95%)	DR (IC95%)	*p* ^a^ value
	Yes		No				
	f	(%)	f	(%)			
Present	154	(69.4)	68	(30.6)	1.72 (1.16–2.54)	12.61 (3.71–21.5)	0.005*
Absent	126	(56.8)	96	(43.2)	Ref.	Ref.	

*OR* odds ratio, *RD* risk difference, *CI 95 %* confidence interval at 95 %

*Statistically significant difference

^a^Chi-square test

Table 3 describes the bivariate association between the covariates and course completion. We observed a higher completion percentage among women (67.7 %) than men (52.2 %). Females had a 92 % increase in chance of completion (OR = 1.92; CI 95 % = 1.26–2.90; *p* = 0.001). An age analysis showed that students aged 30 to 39 have a 40 % lower chance of completing the course than students between 22 and 29 years of age (OR = 0.60, CI 95 % =0.38–0.94; *p* = 0.025). With respect to professional training, the chance that a doctor would complete the course was 57 % lower than the chance that a nurse would (OR = 0.43; CI 95 % = 0.23–0.82; *p* = 0.011).

**Table 3:** Measures of association between covariates and failure to complete the course

Variables	Course completion				OR (95 % CI)	*p* ^a^ value
	Yes		No			
	F	(%)	f	(%)		
Gender						
Female	210	(67.7)	100	(32.3)	1.92 (1.26–2.90)	0.001*
Male	70	(52.2)	64	(47.8)	Ref.	
Age						
22–29	151	(66.8)	75	(33.2)	Ref.	
30–39	69	(54.8)	57	(45.2)	0.60 (0.38–0.94)	0.025*
40–49	38	(67.9)	18	(32.1)	1.04 (0.56–1.95)	0.881
50 and up	22	(61.1)	14	(38.9)	0.78 (0.37–1.61)	0.502
Professional training						
Nursing	158	(63.7)	90	(36.3)	Ref.	
Odontology	102	(68.0)	48	(32.0)	1.21 (0.78–1.86)	0.383
Medicine	20	(43.5)	26	(56.5)	0.43 (0.23–0.82)	0.011*
Marital status						
With a partner	90	(60.4)	59	(39.6)	0.84 (0.56–1.26)	0.409
Without a partner	190	(64.4)	105	(35.6)	Ref.	
Years since graduation completion						
Less than 2 years	55	(68.7)	25	(31.3)	1.29 (0.69–2.43)	0.414
2 to 5 years	111	(60.3)	73	(39.7)	0.89 (0.54–1.48)	0.675
6 to 10 years	53	(63.9)	30	(36.1)	1.04 (0.56–1.91)	0.893
More than 10 years	61	(62.9)	36	(37.1)	Ref.	
Previous post graduate						
Yes	50	(64.1)	28	(35.9)	1.05 (0.63–1.75)	0.834
No	230	(62.8)	136	(37.2)	Ref.	
Professional place of work						
In the country	167	(65.0)	90	(35.0)	1.21 (0.82–179)	0.326
São Luís (Capital)	113	(60.4)	74	(39.6)	Ref.	

*OR* odds ratio, *CI 95 %* confidence interval at 95 %

*Statistically significant difference

^a^Chi-square test

Table 4 presents the analysis of the multivariate logistic regression model to obtain the adjusted *odds ratio*. After statistical adjustment, the variable presence of the monitoring system remained in step with course completion (adjusted OR = 1.74; CI 95 % = 1.17–2.59; *p* = 0.005).

**Table 4:** Multivariate logistic regression analysis of variables associated with completion rate

Variables	Adjusted OR^a^	(CI 95 %)	*p* value
Monitoring System	1.74	(1.17–2.59)	0.005*
Female	1.86	(1.20–2.86)	0.004*
Age 30 to 39 years	0.65	(0.41–1.03)	0.072
Doctors	0.59	(0.28–1.24)	0.169

*OR* odds ratio

*Statistically significant difference

^a^Adjusted regression model for the variables: age group, monitoring system, profession and gender

## Discussion

The purpose of our research was to demonstrate the effectiveness of a monitoring system in the DE modality. As shown in Table 2, the research shows that Monsys contributed to a statistically significant reduction in the dropout rate of approximately 12.6 %. The gender, age and students’ professional training variables also emerged as factors affecting course completion. By contrast, year of graduation completion, whether there is previous postgraduate education, marital status and the student’s professional place of work (in the capital or in the country) did not correlate with the rate of course completion.

We also observed that the highest percentage of matched samples consisted of females (69.8 %). This finding corroborates the findings of Morais et al. [22], Silva et al. [23], Coutinho et al. [24] and Nicholas [25], showing that women make up the majority of students in DE courses.

By contrast, a higher dropout rate among adult female students is described by McGiveny [26]. For this author, the phenomenon results from a combination of family commitments and lack of spousal and/or family support, which creates a conflict between the roles of student, housewife and professional. The results presented in this paper do not support McGivney’s vision but instead corroborate the larger body of literature, showing that in addition to comprising more than two-thirds of the students, women had a 92 % greater chance of completing the course than men (67.7 % versus 52.5 %). This result suggests that when there is an effective and systematic pedagogical supervision of students, such as with Monsys, female students tend to remain more participatory and successfully complete their studies.

Pallof and Pratt [27] argue that the best pedagogical practice in online education is student-centered, which can lead to a reduced dropout rate. Those responsible for organizing DE must be sensitive to students who have learning problems or difficulties with technological instruments and must seek to promote greater interaction such that these students feel recognized as people and not just as virtual students [28].

With respect to age, Mezzari et al. [14] state that the typical DE student is at a more advanced age, which can contribute to problems with the use of technology as a resource and with support. They also emphasize that younger students tend to be more facile at using technology and therefore can more naturally perform VLE tasks. In the present study, however, a correlation was found between the use of Monsys and a lower dropout rate in the 30–39 year age group.

Maciel et al. [29] argue against the claim that older students prefer DE, reporting a trend of younger groups of post-graduate students in DE courses. Yukselturk and Top [30] and Dillie [31] also state that the dominant profile of students in DE no longer consists of individuals over 30 years old and post-graduate students and is becoming a more heterogeneous population with differences in gender and age. This divergence in the predominant age of the typical DE student echoes what Ferreira et al. [32] observed before concluding that there is no defined age group for students attending DE courses and that age is therefore not a fixed variable. The results of our research confirm the trend of younger students in the DE modality, with a predominance of students in the 22–29 year age group.

With regard to professional profiles, nurses comprise the largest share of the students (55.9 %) in the matched samples. These data confirm a trend found in UNA-SUS/UFMA, in which most students are nurses. Since its foundation in 2010, UNA-SUS/UFMA has enrolled a total of 5883 students [33]. Of these, only 2572 students provided information regarding their academic backgrounds, and 1225 enrollees indicated nursing training on their enrollment form [34].

To better understand this trend, we reviewed the research on higher education courses registered with the Ministry of Education (MEC). In Brazil, there are 784 nursing graduation courses, compared with 223 medicine courses and 219 odontology [35] courses. This discrepancy points to a higher number of nurses in the labor market, which would also explain the fact that these students are in the majority at UNA-SUS. Another factor is that a smaller number of residency options in the nursing field leads to the demand for specialized courses.

The high demand among nurses for specialization in Family Health in the DE Modality may also be attributable to the number of job offers by the Family Health Program (FHP) throughout Brazil. According to Ministry of Health data, the positions offered by the program in 2011 accounted for 11.8 % of the formal job offers for graduates in nursing.

Finally, analysis of the impact of the usage of Monsys in each of the categories studied revealed that its best performance was associated with the following variables: presence of the monitoring system and female gender, both of which remained correlated with course completion.

The positive results obtained in this study can be augmented by other studies that address issues such as the influence of the use of Monsys and the participation and implementation of didactic sequences by tutors, thereby allowing for new qualitative projects.

## Conclusion

This study contributes to the reduction of dropout in DE, a problem that affects people and organizations, bringing personal, social and economic waste. The matched sample of 2011, displaying the same conditions as the matched sample of 2010, maintained a conclusion rate of nearly 70 %. This result suggests that efficient pedagogical monitoring is a determining factor in reducing the dropout rate, independent of external factors affecting the process of teaching and learning, including age, gender and profession of the students.

This finding suggests the importance of the pedagogical monitoring made possible with data collected through the use of the Monsys monitoring system, which enabled the pedagogical teams to track the performance of students and tutors in the VLE, accompanying them in their construction of knowledge processes.

It is worth mentioning that, because this is a retrospective study, it was not possible to achieve a more qualitative approach, considering the point of view of students and tutors of the sample groups. In addition, the results can not be automatically extrapolated to other institutional realities, since this would require more tests and applications of Monsys in new and varied contexts, something we intend to do within the continuity of this study.

Finally, the pedagogical monitoring made possible with Monsys subverts the logic that it is the student who must fit the institution, adopting strategies to be successful and adapting to the institutional culture. Instead, this study supports the notion that educational institutions also have the responsibility to adjust to different types of students, promoting quality education for all.

## Abbreviations

ABRAEAD: Brazilian Statistical Yearbook of Distance Education
DE: Distance education
FHP: Family Health Program
ICTs: Information and communication technologies
INEP: Brazil’s National Institute of Educational Studies and Research
INPI: National Institute of Industrial Property
MEC: Ministry of Education
Moodle: Modular Object-Oriented Dynamic Learning Environment
SGTES: Secretary of Labor Management and Health Education
SUS: National Health System
UFMA: Federal University of Maranhão
UNA-SUS: Open University of Brazilian National Health System
VLE: Virtual learning environment
